# Mitigating circumstances: A model-based analysis of associations between risk environment and infrequent condom use among Chinese street-based sex workers

**DOI:** 10.1371/journal.pone.0195982

**Published:** 2018-05-15

**Authors:** Ruth C. Chang, Katie Hail-Jares, Huang Zheng, Na He, Jennifer Z. H. Bouey

**Affiliations:** 1 Department of International Health, School of Nursing and Health Studies, Georgetown University, Washington DC, United States of America; 2 Griffith Criminology Institute, Griffith University, Mt. Gravatt, Queensland, Australia; 3 Shanghai CSW&MSM Center, Xinjian St., Shanghai, China; 4 Department of Epidemiology, Fudan University, Wu Jiao Chang, Yang Pu Qu, Shanghai, China; Old Dominion University, UNITED STATES

## Abstract

**Background:**

Little is known about how freelance street-based sex workers navigate condom use while soliciting. Traditional behavioural model may fail to account for the complex risk environment that most street-based sex workers work within. We examine first the association of self-efficacy and the infrequent condom use, then we investigated the roles of clients and venues frequented on this association.

**Method:**

Using a purposive chain-referral sampling method, we surveyed 248 street-based sex workers in Shanghai. The survey focused on sex workers HIV risk factors, sex work patterns, HIV knowledge, and related HIV self-efficacy. Clients types and behaviours, and characteristics of the venues frequented by these commercial sex workers were also collected. We conducted a series of multiple logistic regression models to explore how the association between a sex worker’s self-efficacy with infrequent condom use change as client and venue characteristics were added to the models.

**Results:**

We find that within the basic model, low self-efficacy was marginally associated with infrequent condom use (54.9% vs. 45.1%, AOR = 1.70, 95% CI = 0.95–3.03). As client- and venue- characteristics were added, the associations between self-efficacy and condom use were strengthened (AOR = 2.10 95% CI = 1.12–3.91 and 2.54 95% CI = 1.24–5.19 respectively). Those who reported middle-tiered income were more likely to report infrequent condom use compared to their peers of high income (AOR = 3.92 95% CI = 1.32–11.70) whereas such difference was not found between low income and high income sex workers. Visiting multiple venues and having migrant workers as clients were also associated with infrequent condom use.

**Conclusion:**

Our findings suggest sex worker’s self-efficacy matters in their HIV risk behaviours only when environment characteristics were adjusted. Risk environment for street-based sex workers are complex. Programming addressing behavioural changes among female sex workers should adopt holistic, multilevel models with the consideration of risk environments.

## Introduction

By the end of 2014, over 501,000 people were living with HIV/AIDS in China [1]. While the overall prevalence rate remains low (0.037%), and the number of new diagnoses continue to decline, the burden of HIV/AIDS in China has shifted. Increasingly, sexual activity rather than injection drug use has become a major route for HIV/AIDS transmission [1]; heterosexual activity accounted for just 30% of new HIV cases in 2005 growing to over 66% in 2014 [1–3]. As with other countries, this shift to sexual transmission has led to the “feminization” of the virus with women comprising one of the fastest growing demographics among new infections [4–7]. At the same time, as new incidents of HIV decrease overall, other sexual transmitted infections (STI) rates have rapidly increased in China, following the same shift in transmission patterns [8–11]. Though HIV rates have remained relatively stable for Chinese female sex workers since 2005, the government has increasingly seen this population as a group necessitating greater scrutiny and as a conduit for improving sexual education among their male customers [8,12–15]. Intervention programs for female sex workers, then, have primarily focused on increasing rates of (male) condom use through education and fostering more self-efficacy through assertiveness training or economic investment [14–23].

Such programs almost exclusively draw upon the philosophical tenants of the information-motivation-behavioural skills (IMB) model [24–28]. The IMB model suggests that risky sexual behaviours occur in the absence of three characteristics: 1) knowledge about HIV/STI transmission; 2) confident prevention-related behaviour (such as knowing how to negotiate condom use as well as properly use the device); and 3) individual motivation to prevent transmission. The simplicity of the IMB model—that education, empowerment, and motivation can effectively prevent the spread of HIV/STIs—is attractive both in terms of directing programs, but also evaluation [25]. The various aspects of the IMB are easily quantifiable. Evidence-based assessments have shown that the IMB model can successfully be used with a range of populations, settings, and locations [29–34].

However, recent criticism of the IMB has underscored that it may be too simplistic and fails to account for larger environmental factors [26, 27, 35]. In such critiques, the focus on the outcome obscures how other environmental factors, such as culture, criminalization, venues, and client relationships, mitigate both the individual-level risk factors *and* the eventual resulting behaviour (such as condom use). Building upon earlier work, Yang and Xia constructed a model that incorporated social environment mediators into a traditional IMB model framework [36]. Relying upon a sample of lower-level indoor female sex workers, Yang and Xia incorporated measures for group norms, peer pressure, and venue characteristics through a series of multivariable logistic regression models. They concluded that as more complicated models were constructed, venue support for condom use lost its significance, instead mitigated by self-efficacy and peer support. Such a finding suggests that factors that seem important early in a modelling process may lose their impact on risk behaviours as more complicated ecological models are constructed [36].

Yet, the Yang and Xia study relied upon indoor venue-based female sex workers in China and a theoretical understanding of how social support and venue characteristics may impact their risk behaviours. These same findings are unlikely to be generalizable to street-based female sex workers (SBSWs) who work without a manager nor a particular affiliation with an entertainment venue for client solicitation. These sex workers account for a disproportional burden of STIs in China [37,38] and were 2.7 times more likely to test positive for HIV compared to sex workers from middle- and higher-tier venues [38]. Since they do not have an established affiliation with a managed venue, they are highly mobile among different venues, have diverse clientele, and an elevated risk of arrest or involvement in street violence [39–41]. Previous research has noted that they have smaller social support systems (both with peers and with family or friends), are often older, and more likely to be migrants than those who worked exclusively at managed indoor environments, such as karaoke bars (KTV), hair salons, or massage parlours [42]. Within these circumstances, the same peer and venue-based social support systems that were so important to indoor sex workers may not be as important. In this paper, we examine the association of client characteristics and venue environment on condom use and self-efficacy among street-based female sex workers.

### Client characteristics

Male clients engaged in transactional sex can play a crucial role in a female sex worker’s ability to successfully use a condom. Perhaps unsurprisingly, the same factors that determine condom use among female sex workers have also been identified as significant predictors of client condom use. Notably, greater self-efficacy, greater awareness of STI risk, and previous education on safer sex practices were all associated with higher rates of condom use among clients [33,43]. Unfortunately, research that relies upon surveys with sex workers may not be able to accurately capture this information; sex workers simply may not know whether their clients have participated in sexual education.

Other studies have noted that client type often serves as an indicator of client socio-cultural background. However, such distinctions have yielded mixed results. Safika, Levy and Johnson found that Indonesian female sex workers reported more infrequent condom use with foreign rather than domestic clients [44]. Qualitative research among Nepalese sex workers found the same association [45]. When socio-economic class signifiers were added to descriptions, further breakdowns were noted. In their qualitative research on barriers to condom use, Jie, *et al*., found that Chinese sex workers explained they were more suspicious of migrant laborers and, in turn, more likely to use condoms with them as opposed to white collar workers [46]. Thus, sociodemographic signifiers of education and “cleanliness”, and xenophobia may combine to mitigate condom use and sex worker self-efficacy in such situations.

### Venue environment characteristics

Commercial sex in China is illegal but widespread [13,47]. The sheer size of the commercial sex industry in China has contributed to the diversity of sex workers available. Female sex workers themselves range from college students who work as part-time, street-based courtesans and escorts, brothel-based sex workers, to older streetwalkers [26]. One study lists twelve different types of female sex workers reflecting a variation of work organisations, incomes, and clientele [48]. The growing number of female sex workers has also caused the price of commercial sex to plummet, increasing economic pressure on lower-income sex workers [49].

Working indoors has consistently been shown as a protective factor against HIV/STI infections [50–52]. In most cases, risk reduction has been tied to in-house or managerial policies regarding condom use [27, 53–55]. Indeed, in their comparison of SBSW to venue-based sex workers, Yi and his colleagues found that SBSW were much more likely to be self-reliant and expressed frustration or despair over not having the helpers when faced with difficult or violent clients [50]. However, this lack of place-based employment does afford SBSW a certain independence to move between various indoor venues (such as bars and cabarets) [56]. Bharat and her co-authors identified autonomous mobility—the ability to self-select one’s workplace—as predictive of regular condom use and may enhance self-perception of self-efficacy [18].

**Fig 1:**
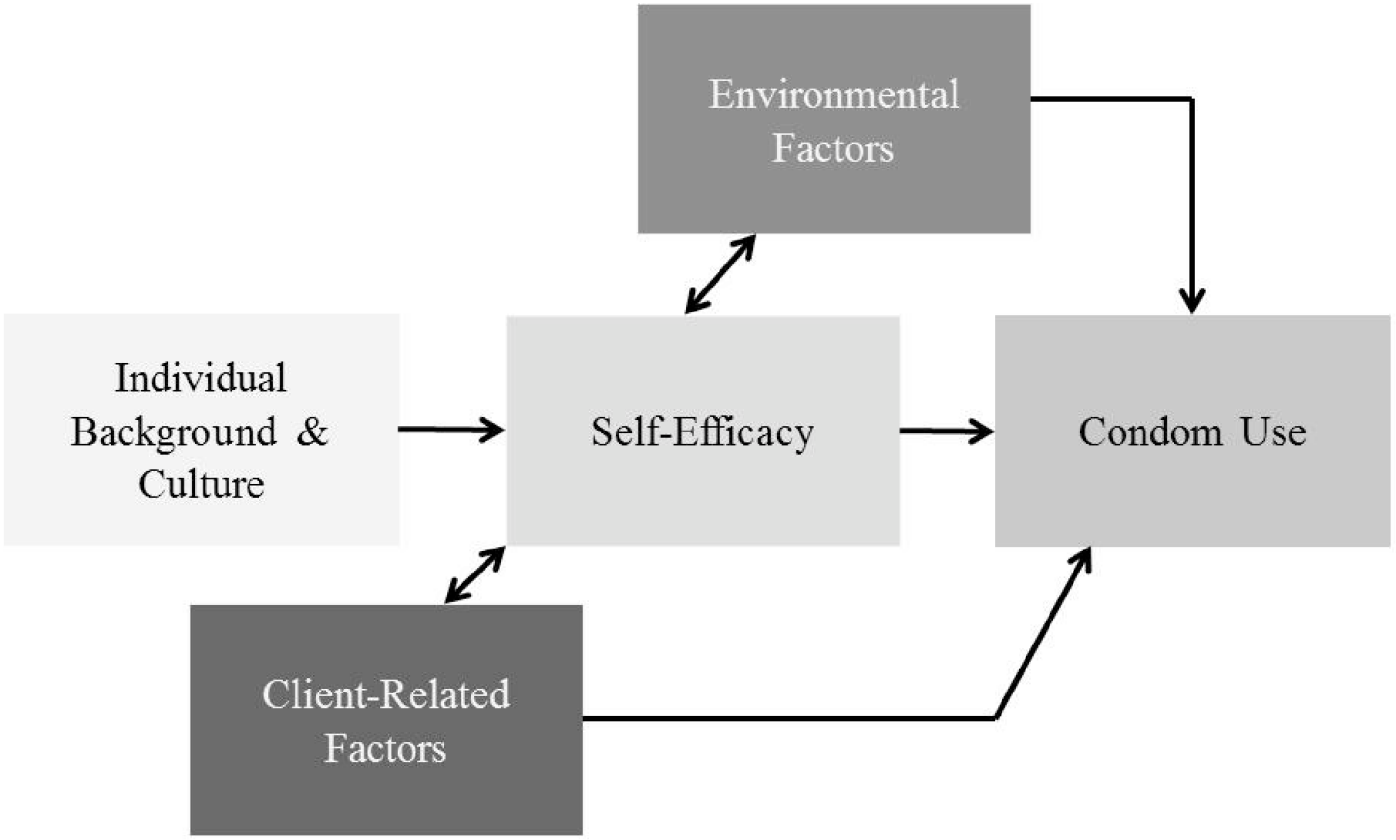
Theoretical map of factors influencing condom use among street-based sex workers.

Here, we consider the association of client- and venue-level characteristics on SBSW’s condom use, as well as examine how such factors mitigate individual-level factors relating to personal autonomy, notably self-efficacy (Fig 1). Through a series of multi-variable logistic regressions, we examine two research questions: 1) Are client- and venue-level characteristics associated with SBSW’s condom use? and 2) How client and venue characteristics mitigate the influence of individual characteristics (income and self-efficacy) on the infrequent condom use?

## Materials and methods

### Study setting and population

This analysis was part of a larger study examining the lives and working conditions of older SBSWs in Shanghai, conducted between 2011 and 2012. Shanghai was selected since the city has been a gateway to rural-to-urban migration. Migrants account for nearly 40 percent of the city’s population of 23 million [57]. The migration has generated an increase in the city’s sex work sector, both in the number of buyers and sellers [58–59]. Migration, the commercial sex industry, and continued drug trafficking within Shanghai have led to the regionalization of sexually transmitted infections in the area, and, within the city, rising rates of HIV/AIDS and syphilis [59–61].

All participants were recruited from the Zhabei District which hosts the Shanghai railway station, the main point of entry for migrants in the city. Researchers, with the help of staff members of Shanghai CSW&MSM Center, a non-government organization (NGO) that serves HIV high-risk populations, identified seeds during the qualitative data collection phase before the survey study. Eight street-based sex workers were recruited to serve as “seeds” for this study (4 migrants and 4 non-migrants). A snowball recruitment process was used, and each seed and their subsequent recruits were asked to refer three other SBSW to participate in the study. Eligible participants (including seeds) needed to be: (1) biologically female; (2) 18 to 65 years of age; (3) able to provide verbal or written consent in Mandarin; (4) self-identified as a commercial sex worker (having sex with men for money or goods) at the time of the survey; and (5) unmanaged (unaffiliated with a brothel or other indoor location) and primarily street-based in their solicitation of clients.

Surveys and interviews were conducted according to the participant’s discretion in either a public cafe or in the office of Shanghai CSW&MSM Center. Each participant who finished the questionnaire was also given three coupons to distribute to the next wave of recruits. Each coupon had a unique code linking the recruit to her recruiter. When the referee finished the survey, the recruiter received a $5USD reimbursement incentive. All interview instruments and study protocols were approved by the Institutional Review Boards of both Georgetown University and Fudan University. Each participant received $15 USD in cash as compensation for their time and travel expenses, as well as a pre-packaged health education resource kit.

### Measurements

#### Primary outcome: Condom use

Our primary outcome variable is the frequency of vaginal condom use with clients (oral condom use was almost non-existent in this survey population). Participants were asked “How often do your customers use condoms during vaginal sex?” and provided with the possible responses of a) Never; b) Once in a while; c) Sometimes; d) Most of the time; or e) Always. Based upon responses, we created a dichotomized condom use variable: “infrequent condom use” (Options a-c) and “frequent condom use” (Options d & e).

#### Independent variables

**Individual Characteristics**

Our focus for individual-level factors was to have them serve as a proxy for personal autonomy. Sociodemographic information from SBSW respondents, including age, education-level, monthly income, residency (migrant or non-migrant), proficiency in the Shanghainese dialect (as self-reported), and marital status were collected. In addition to sociodemographic factors, self-efficacy was considered an individual-level factor. Self-efficacy is an individual’s perception of their ability to complete a behaviour. Within IMB models, self-efficacy is often associated with one’s ability to successfully advocate for condom use with their partners. Self-efficacy regarding condom use was measured using a six-item scale that is similar to previously used scales in FSW research in China [27, 61–62]. Respondents were asked to rank the following statements on a Likert scale ranging from strongly disagree (1) to strongly agree (5): a) I can ask a new partner to use condoms; b) I can discuss practicing safe sex with my partner; c) I can discuss practicing safe sex with a client; d) I can refuse sex when a partner does not have a condom; e) If a partner does not want to use a condom, I can persuade him; and 6) If a client does not want to use a condom, I can persuade him. An average index of self-efficacy was then created, with the highest score of 5 and the lowest of 1 (Cronbach’s α = 0.84). The variable was then split at the median (3.83). We then defined “low self-efficacy” as less than 3.83” and “high self-efficacy” as equal to or greater than 3.83.

**Client Characteristics**

Since education and cultural differences can influence condom use behaviour, providing basic demographic information about clients was helpful. Respondents were asked “In the last six months, your clients were mostly (a) Shanghai locals; (b) Chinese non-local businessmen; (c) migrant workers; (d) foreigners; or (e) other.” SBSWs were also asked how frequently, on a weekly basis, they had clients who were under the influence of alcohol. The same response options as provided for condom usage were provided here, and the same aggregation occurred (frequently/infrequently).

**Venue Characteristics**

Participants were asked where they solicited customers. Options included outdoors, such as on the street or in parks, or indoors, including in bars, MTV cabaret. As noted, SBSW in Shanghai may solicit clients from multiple venues including indoors, but are notably not tied to a manager. Our sample of SBSW also noted they found clients through peer referral and maintained contact by phone (used to arrange meetings with repeat, regular clients). Respondents could check all that applied. We then further aggregated venue by the type of environment where solicitation occurred (outdoor, indoor, or both) and the total number of types of venues (low-, middle- and high-end venues) in which a sex worker used (one, two, three or more).

### Statistical analysis

Responses from 248 subjects were included in the analysis. Bivariate analysis using chi-square (categorical variables), Fisher test of exact variance (for categorical variable with cell count under 5), and two-way ANOVA (for continuous variables) were used to analyse the association between individual, client, and venue-level characteristics and condom use. Following the bivariate analysis, variables that were both in our theoretical framework (individual-, client-, and venue- characteristics) and statistically significant were included in a series of multivariable logistic models (Fig 1). The first model included selected individual characteristic variable only, the second model included an additional variable of client type, the third model include two venue characteristics. To prevent overfitting and avoid multiple -collinearity, we only limited the models to two variables that were not highly correlated per each theoretical group (demographics, individual HIV, clients, and venue). Other than self-efficacy, age, income, and language (proxy for migrant status) were included in the model as the individual level characteristics. With the client characteristics, we were only able to choose one variable although both variables were significant because very few SBSW reported their clients were drunk (n = 20). The adjusted odds ratios were calculated for each independent factor of individual-, client-, and venue- characteristics, as it related to condom use. The models’ overall changes in explanatory value was also recorded. All statistical analysis was carried out in SAS 9.4 (Cary, NC).

## Results

### Individual-level factors

Demographically, over half of our sample were 32–37 years old (60%), migrants (94%), and most likely to have no education or at least an elementary education (60.7%). However, these demographic characteristics—age, residency status, and education level—were not significantly associated with condom use (Table 1). Low self-efficacy was marginally associated with infrequent condom use. Among those respondents with low-self efficacy, nearly 55 percent reported infrequent condom use compared to just 43 percent of those with high self-efficacy (p = 0.068; Table 1). Beyond self-efficacy, demographic factors related to personal autonomy, such as language proficiency, monthly income, and marital status were related to condom use, though not necessarily in ways that followed expected patterns. Infrequent condom use was more likely to be reported by individuals who had perfect language proficiency (52.2%) than those that rated their language skills as at least “good” (30.2%) (p = 0.01; Table 1). Similarly, monthly income formed a bell curve, with sex workers at the two income extremes (<1000 per month and >5000 per month) both reporting frequent condom use over 70 percent of the time (p = 0.02; Table 1), while mid-level earners (1000.01–4999.99 per month) more often engaged in infrequent condom use. Finally, respondents who were single and had never married reported the highest rate of frequent condom use with clients (81.8%), a larger percentage than the rate reported by widowed or divorced respondents (53.6%) or married respondents (48.0%) (p = 0.09; Table 1). Although, given there were only 11 singles in the study and with a p-value greater than 0.05, we caution in interpreting the last point as a significant finding.

**Table 1:** Self-efficacy and demographic characteristics and their associations to condom use among street-based female street based sex worker, Shanghai, China.

Variable	N = 248	Condom Use with Clients % (N = 248)		Chi-Square P-Value
		Infrequent (n = 119)	Frequent (n = 129)	
**Self-efficacy**				*0*.*068*
Low Self-efficacy	41% (102)	54.9% (56)	45.1% (46)	
High Self-efficacy	59% (146)	43.2% (63)	56.8% (83)	
**Age**				0.43
20–25 years old	10% (25)	40.0% (10)	60.0% (15)	
26–31 years old	17% (41)	58.5% (24)	41.5% (17)	
32–37 years old	60% (148)	46.0% (68)	54.0% (80)	
38+ years old	13% (32)	46.9% (15)	53.1% (17)	
**Residency**				0.58
Migrant	94% (222)	46.0% (102)	54.0% (120)	
Non-migrant	6% (15)	53.3% (8)	46.7% (7)	
**Education Level**				0.44
None/Elementary	60.7% (141)	48.9% (69)	51.1% (72)	
Middle School//High School/College	39.3% (91)	43.9% (40)	56.1% (51)	
**Language (Shanghai Dialect)**				***0*.*01***
Cannot speak/Speak very little/Good	**19% (43)**	**30.2% (13)**	**69.8% (30)**	
Excellent	**81% (180)**	**52.2% (94)**	**47.8% (86)**	
**Monthly Income (RMB)**				***0*.*02***
≤1000	**6% (14)**	**28.6% (4)**	**71.4% (10)**	
1000.01–2999.99	**37.5% (93)**	**51.6% (48)**	**48.4% (45)**	
3000–4999.99	**41.5% (103)**	**54.4% (56)**	**45.6% (47)**	
≥5000	**15% (37)**	**29.7% (11)**	**70.3% (26)**	
**Marital Status**				*0*.*09*
Never Married	5% (11)	18.2% (2)	81.8% (9)	
Married	50% (123)	52.0% (64)	48.0% (59)	
Divorced/Widowed	45% (110)	46.4% (51)	53.6% (59)	

### Client-level factor

Sex workers who primarily served Shanghai reported the lowest rates of infrequent condom use at 25 percent (Table 2). Comparably, sex workers who served migrant workers or non-local businessmen reported that they most often engaged in infrequent condom use, with 56.5 and 53.6 percent respectively (p = 0.0014; Table 2).

**Table 2:** Client and venue characteristics by condom use status among street-based sex workers in Shanghai, China.

		Condom use with clients % (N = 248)		Chi-Square P-Value
	N = 248	Infrequent (n = 119)	Frequent (n = 129)	
Regular contact with inebriated client (weekly)*				***0*.*0004***
Infrequent	**92% (227)**	**51.5% (117)**	**48.5% (110)**	
Frequent	**8% (20)**	**10.0% (2)**	**90.0% (18)**	
**Type of clients**				***0*.*0014***
Shanghai local	**21.3% (51)**	**25.5% (13)**	**74.5% (38)**	
Non-local businessmen	**69.2% (166)**	**53.6% (89)**	**46.4% (77)**	
Migrant workers	**9.5% (23)**	**56.5% (13)**	**43.5% (10)**	
**Venue Environment**				***0*.*02***
Outdoor	**54.8% (136)**	**55.2% (75)**	**44.8% (61)**	
Indoor	**30.6% (76)**	**43.4% (33)**	**56.6% (43)**	
Both	**14.6% (36)**	**30.6% (11)**	**69.4% (25)**	
**Number of Venues Served**				***0*.*0001***
1 venue	**12.1% (29)**	**31.0% (9)**	**69.0% (20)**	
2 venues	**29.3% (70)**	**21.4% (15)**	**78.6% (55)**	
3+ venues	**58.6% (140)**	**66.4% (93)**	**33.6% (47)**	

*Despite its statistical significance, we omitted this variable from the multivariable models due to its low extreme count (<5) in one of the cells. Hence, we were able to only include one client-level factor in the multivariable models.

### Venue-level factors

Venue factors were statistically significant in their association with condom use (Table 2). Unsurprisingly, a large proportion of the SBSW worked either outdoors or in both indoor and outdoor environments. Those respondents who indicated they worked both indoors and outdoors reported the highest rate of frequent condom use, about 70 percent, compared to those that worked indoors-only (57 percent) or outdoors-only (45 percent). Flexibility in where a sex worker solicited clients, thus, was associated with higher rates of reported condom use (p = 0.02; Table 2). Similarly, number of venues served was also highly correlated with reported client condom use, with only a third of those working at three or more venues reporting frequent condom use compared to 69 percent and 79 percent among those who worked at one or two number of venues (p = 0.0001; Table 2).

### Multivariable level models

Once the bivariate analyses were completed, we wanted to further examine how the role of self-efficacy changed as each level of factors was added to the model. Model #1 included individual level factors (self-efficacy, language proficiency, and monthly income; Nagelkerke Pseudo-R^2^ = 0.07) in predicting infrequent condom use when controlled for age (Table 3). Within this first model, low self-efficacy was not significantly associated with infrequent condom use, but sex workers earning high mid-level income (3000–4999.99 per month) were approximately 2.5 times more likely to infrequently use condoms than the highest earning workers (OR 2.51; 95% CI: 1.01–6.28; Table 3).

**Table 3:** Adjusted ORs and the 95% confident intervals of the individual-, client-, and venue-level factors on the street-based sex workers’ infrequent condom use with clients in Shanghai, China.

Variable	Model #1: Individual Level Factors	Model #2: Individual + Client Level Factors	Model #3: Individual + Client + Environmental Level Factors
**Individual Factors** ^a^	**(n = 221)**	**(n = 215)**	**(n = 208)**
**Self-Efficacy-condom use (reference: high self-efficacy)**			
Low self-efficacy	1.70 (0.95–3.03)	**2.10 (1.12–3.91)**	**2.54 (1.24–5.19)**
**Language—Local dialect (reference: excellent in local dialect)**			
Poor/Good	0.47 (0.22–1.02)	0.67 (0.29–1.50)	1.03(0.49–2.78)
**Monthly Income (reference:>5000RMB)**			
<1000	0.78 (0.16–3.77)	1.53 (0.28–8.31)	2.64 (0.41–17.04)
1000.01–2999.99	2.43 (0.96–6.176)	**3.03 (1.14–8.50)**	**5.34 (1.70–16.76)**
3000–4999.99	**2.51 (1.01–6.28)**	**3.14 (1.12–8.25)**	**3.92 (1.32–11.70)**
**Client Factors** ^b^			
**Type of clients (reference: local residents)**			
Non-local businessmen		4.16 (1.76–9.82)	2.47 (0.84–7.21)
Migrant workers		**5.39 (1.61–18.11)**	1.86 (0.44–7.79)
**Venue Factors** ^c^			
**Venue Environment (reference: Both outdoor and indoor)**			
Outdoor			2.15 (0.69–6.67)
Indoor			2.79 (0.78–10.00)
**Venue Stability (Number of Venues Served) (reference: 1 venue)**			
2 venues			0.54 (0.16–1.82)
3 venues or more			**4.40 (1.41–13.67)**
**Model-Level Information**			
Nagelkerke Pseudo-R^2^	0.11	0.17	0.34
F *(df)*	8	10	14
*p*-value of model	0.02	0.0008	<0.001

^a^ Within *Individual-level* factors, the following reference categories were used: high self-efficacy, perfect language skills, and monthly income of 5000.00 or greater. All models were controlled for age.

^b^ Within *Client-level* factors, the following reference categories were used: having Shanghai locals as clients.

^c^ Within *Venue-level* factors, the following reference categories were used: working both indoor and outdoor venues and serving just one venue.

We then added a client-level factor—types of clients—to the previously listed individual-level factors. The additional factor increased the explanatory value of the model (Nagelkerke Pseudo-R^2^ = 0.16). Once the client-level factor was added, self-efficacy became statistically significant. Sex workers who reported low self-efficacy were 2.16 times more likely to also report infrequent condom use (OR 2.09; 95% CI: 1.12–3.91; Table 3). Similarly, the odds of infrequent condom use among high mid-level earners grew slightly (3.03; 95% CI: 1.14–8.50). Sex workers whose clients were mostly migrant worker or foreigners were 5.9 times more likely to report infrequent condom use than those SBSWs that primarily served Shanghai locals (Table 3).

The association of client-level factors was lessened, rendering them insignificant, once venue-level factors were added to the final model, Model #3. In Model #3, both the venue environment and total number of venues served were added to the variables included in Model #2. As before, the addition of the venue-level factors further increased the explanatory value of the model (Nagelkerke Pseudo-R^2^ = 0.34). In this complete model, self-efficacy gained yet more significance, with sex workers with low self-efficacy nearly three times as likely to report infrequent condom use (OR 2.54; 95% CI: 1.24–5.91; Table 3). The inclusion of venue factors enhanced the odds that high-mid-level earners would engage in infrequent condom use. In the final model, high mid-level earners were four times more likely to engage in infrequent condom use than the highest earners. Also, within Model #3, the difference between low mid-level earners was further exacerbated; those earning 1000.01–2999.99 per month were 5.3 times as likely to report infrequent condom use when compared to the reference group. None of the venue-level factors emerged as statistically significant.

## Discussion

Previous behavioural modelling with multi-level factors has relied upon samples of venue-based sex workers. Comparatively, street-based sex workers operate within a more exposed and often unstructured environment. In regard to our first research question, we expected that client-based factors and venue factors, specifically the ability to work across different types work environments, would mitigate how SBSW self-efficacy potentially affect frequent condom use. In the presence of both client and venue-related factors, low self-efficacy was associated with a three times greater likelihood of infrequent condom use when compared to the initial model. That initial model, which considered self-efficacy only with adjustment of demographic variables, suggested that on its own, without inclusion in the broader risk environment, the ability to advocate for condom use was not significantly associated with condom use. For our second research question, our findings are further supported by the importance of SBSW income in all three models and suggest that economic pressure may serve as an objective check to the more subjective measure of self-efficacy, when considering individual sexual worker autonomy.

### Individual-level factors

Modelling that incorporates an ecological theory of sex work further stresses how additional human and environmental interaction increases the importance of self-efficacy to reducing risk behaviour, but also indicates the specific moderating factors for the effect of self-efficacy. However, these client- and environmental-level factors were not the only ones that had bearing on condom use. In each iteration of the model, a single socioeconomic factor remained statistically significant—overall income of sex workers at both extremes—the very low earners and very high earners—expressed greater likelihood to frequently use condoms with clients. Though literature has focused on the role of greater income in improving autonomy and condom use, less is known about low-end earners. One theory is that such low-income sex workers do exercise more autonomy over client choice. Low-end SBSW does not provide an incentive for pimping or management, and, as such, low-end SBSW may actually exercise more control over client selection than mid-range workers [18].

Another possible explanation—and limitation—is that our original question asked how much respondents made from sex work each month. In a bivariate analysis, 78% of SBSWs reported other type of supplemental income and therefore reported lower income form sex work. Since these part-time workers may be less dependent upon sex work as a source of total revenue, their actual autonomy may be greater, despite appearing here as low-income earners. Disparities in earning may also be linked to client flow. Though we do not present that information here, 85.7% of the lowest earners saw less than the average number of clients a week, compared to 61.8% of all others sex workers. Comparably, among the highest earners, only a third (32.4%) saw a lower number of clients, and two-thirds saw more than 13 (the average) clients a week. The findings were statistically significant (p<0.001) in both scenarios. Future surveys should distinguish between these two earning streams and seek to better understand the role of sex work in participants’ lives.

### Client-level factors

Client-level factors—specifically client type—strengthened the association between self-efficacy and risk behaviour. Having predominantly non-local businessmen as clients was nearly 4.57 times more likely to lead to infrequent condom use when compared to sex workers who mostly served Shanghai locals. Such findings begin to illustrate how clients’ cultural and social beliefs influence, in turn, their sexual partners. These findings also illustrate an opportunity for intervention. Sex workers that work primarily with these two groups should be targeted for programming. Trainings should incorporate examples and role-plays that are culturally specific to these high-risk groups of male clients.

### Venue-level factors

Though the two venue-level factors included in the multivariable modelling—number of venues served and venue environment—were not statistically significant in the multivariable model, their inclusion in the model had notable reverberations, both dissipating the statistical significance of client-level factors and further strengthening the association between self-efficacy and condom use. This impact underscores a methodological concern when relying upon multivariable logistic modelling; such models are highly susceptible to omitted variable bias. Concluding with Model #2 rather than Model #3, for example, would give the false impression that client-level factors are the most important to condom use. Instead, the ability to freely move between types of spaces and work environments had a considerable moderating effect. In the future, public health researchers should endeavour to construct models that include variables from all levels and are reflective of the broader environment that sex workers operate within to control for such omissions [53]. For practitioners, and from an intervention standpoint, the role of venue-level factors suggests venue stability (working at fewer venues) and exercising autonomy in environment choice (working both inside and outside) can lead to more frequent condom use with clients. Notably, like income, the two factors that comprise our venue-level considerations could also serve as more objective measures of overall sex worker autonomy. Among this sample of SBSW, personal autonomy—whether that is measured by income, work environment stability, venue choice, or self-efficacy—led to more frequent condom use.

### Limitations

Compared to many other studies conducted in this area, our sample size was quite small, and this limits the generalizability of our findings. The small sample size resulted in skewed distributions and resulted in extreme confidence intervals for the odds ratios. Although we addressed this issue through collapsing the variables, we may have lost some granularity of data. Furthermore, we were not able to include two variables from level due to the lack of significance. We encourage readers to focus on the methodological approach and use this paper as a quantitative case study that bolsters the argument for conducting ecologically-directed modelling rather than broad generalizable approaches. Next, our modelling focused on two levels of factors (client-level and venue-level) that we theorized as important for SBSW. Future research should incorporate other ecological levels when modelling, including HIV/AIDS knowledge, and social support. Not including these levels of factors may lead to the same omitted variable bias we discuss above. Finally, our study was cross-sectional, which did not allow for casual inferences. However, our paper is less about the specific population and instead demonstrates the importance of understanding how each additional layer of factors act upon one another within a risk environment.

### Conclusion

Building complex multivariable models, without attention to the various types of factors included, can obscure how the various types of variables act upon one another, and, in turn, impact the outcome. Here, we have broken down the model-creation process and rely upon an additive approach to modelling. As client-level and then environmental-level factors were added to the initial model, the statistical significance and substance of individual-level factors, particularly those related to self-efficacy, were strengthened. While these findings suggest that risk environments are complex, we encourage future researchers also to consider and measure higher-level social and political level factors, such as state legal status, individual encounters with the criminal justice system, and extent of social networks. Addressing and incorporating these measures into a risk environment model will further illustrate how systematic reforms can best serve sex workers who face the highest degrees of marginalization.

## Supporting information

S1 Table Edited dataset from the HIV survey of 248 street-based sex workers in Shanghai, China. (XLSX)
